# Optimized Protocol for the In Situ Derivatization of Glutathione with *N*-Ethylmaleimide in Cultured Cells and the Simultaneous Determination of Glutathione/Glutathione Disulfide Ratio by HPLC-UV-QTOF-MS

**DOI:** 10.3390/metabo10070292

**Published:** 2020-07-17

**Authors:** Xueni Sun, Raffaela S. Berger, Paul Heinrich, Ibtissam Marchiq, Jacques Pouyssegur, Kathrin Renner, Peter J. Oefner, Katja Dettmer

**Affiliations:** 1 Institute of Functional Genomics, University of Regensburg, Am BioPark 9, 93053 Regensburg, Germany; xueni.sun@klinik.uni-regensburg.de (X.S.); raffaela.berger@klinik.uni-regensburg.de (R.S.B.); paul1.heinrich@stud.uni-regensburg.de (P.H.); Peter.Oefner@klinik.uni-regensburg.de (P.J.O.); 2 University Côte d’Azur (IRCAN), CNRS-INSERM, Centre A. Lacassagne, 06189 Nice, France; ibtissam.marchiq@gmail.com (I.M.); Jacques.Pouyssegur@unice.fr (J.P.); 3 Department of Medical Biology, Centre Scientifique de Monaco, CSM, 98000 Monaco, Monaco; 4 Department of Internal Medicine III, University Hospital Regensburg, 93042 Regensburg, Germany; Kathrin.Renner-Sattler@klinik.uni-regensburg.de

**Keywords:** glutathione, glutathione disulfide, cell culture, liquid chromatography, UV detection, mass spectrometry

## Abstract

Glutathione (GSH) and glutathione disulfide (GSSG) are commonly used to assess the oxidative status of a biological system. Various protocols are available for the analysis of GSH and GSSG in biomedical specimens. In this study, we present an optimized protocol for the in situ derivatization of GSH with *N*-ethylmaleimide (NEM) to prevent GSH autooxidation, and thus to preserve the GSH/GSSG ratio during sample preparation. The protocol comprises the incubation of cells in NEM containing phosphate buffered saline (PBS), followed by metabolite extraction with 80% methanol. Further, to preserve the use of QTOF-MS, which may lack the linear dynamic range required for the simultaneous quantification of GSH and GSSG in non-targeted metabolomics, we combined liquid chromatographic separation with the online monitoring of UV absorbance of GS-NEM at 210 nm and the detection of GSSG and its corresponding stable isotope-labeled internal standard by QTOF-MS operated with a 10 Da Q1 window. The limit of detection (LOD) for GS-NEM was 7.81 µM and the linear range extended from 15.63 µM to 1000 µM with a squared correlation coefficient *R^2^* of 0.9997. The LOD for GSSG was 0.001 µM, and the lower limit of quantification (LLOQ) was 0.01 µM, with the linear (*R^2^* = 0.9994) range extending up to 10 µM. The method showed high repeatability with intra-run and inter-run coefficients of variation of 3.48% and 2.51% for GS-NEM, and 3.11% and 3.66% for GSSG, respectively. Mean recoveries of three different spike-in levels (low, medium, high) of GSSG and GS-NEM were above 92%. Finally, the method was applied to the determination of changes in the GSH/GSSG ratio either in response to oxidative stress in cells lacking one or both monocarboxylate transporters *MCT1* and *MCT4*, or in adaptation to the NADPH (nicotinamide adenine dinucleotide phosphate) consuming production of D-2-hydroxyglutarate in cells carrying mutations in the isocitrate dehydrogenase genes *IDH1* and *IDH2.*

## 1. Introduction

Glutathione is the major non-protein thiol in mammals, which is present at millimolar concentrations within cells, especially in the liver [1]. In vivo, glutathione exists in a redox equilibrium between its reduced monomeric (GSH) and oxidized dimeric form (GSSG). Glutathione, for the most part, does not react directly with hydroperoxides, but is rather required in a two-step process to reduce the oxidized selenocysteine residues of glutathione peroxidases that catalyze the reduction of hydrogen peroxide and lipid hydroperoxides to water and their corresponding alcohols, respectively [2]. The two molecules of GSH consumed in this process result in the formation GSSG, which is then reduced again to GSH by NADPH in an enzymatic reaction catalyzed by glutathione reductase. The intracellular content of GSSG is normally kept to less than 1% of total glutathione. The GSH/GSSG ratio is widely used as an indicator of the redox state of cells or tissues, with marked decreases indicating oxidative stress, i.e., a biological system’s failure to readily detoxify reactive oxygen species.

Over the years, several methods have been introduced for the determination of GSH and GSSG in biospecimens, including spectrophotometric [3,4,5], UV [6], fluorometric [7,8,9], and mass spectrometric techniques [10,11,12,13], either in batch or in combination with HPLC. In addition to improved detection techniques, increasing attention has been paid to the effective prevention of GSH autooxidation during sample collection and preparation when determining GSH and GSSG. As such, 2-Vinylpyridine (2-VP) and *N*-ethylmaleimide (NEM) are two commonly used derivatizing agents for the analysis of GSH. However, 2-VP suffers from poor cell membrane permeability and slow reactivity with GSH [3]. NEM, on the other hand, is highly cell membrane-permeable and blocks the sulfhydryl group on the cysteinyl residue of GSH quickly by alkylation, thereby stopping GSH autooxidation [14]. In addition, through inhibition of glutathione disulfide reductase (GR), NEM also prevents the enzymatically catalyzed reduction of GSSG [3,15]. Alternatively, an enzymatic recycling method has been reported for the quantitative assay of GSH and GSSG. It involves the reaction of GSH with 5,5′-dithio-bis(2-nitrobenzoic acid) (DTNB) to form UV detectable 5’-thio-2-nitrobenzoic acid (TNB) and the reduction of GSSG to GSH by glutathione reductase and NADPH [4]. This method can be used to determine total glutathione (GSH + GSSG). If the ratio of GSH and GSSG is of the interest, an additional sample aliquot is needed where a -SH masking reagent, e.g., NEM, has to be added in advance. Thus, GSH amount can be determined by subtracting GSSG from total glutathione. Giustarini et al. reported a protocol for GSH and GSSG determination with NEM derivatization in blood and solid tissues [3] and cultured cells [15]. This protocol allows the simultaneous determination of GSH and GSSG. However, GSSG was determined by converting GSSG to GSH with dithiothreitol (DTT) reduction and subsequent fluorescent labeling of the -SH group with monobromobimane (mBrB) for the detection. Moreover, excess NEM has to be removed from the sample prior to DTT reduction and mBrB derivatization to avoid interferences of NEM with the subsequent reduction and derivatization steps which makes the sample preparation complicated and time-consuming. Recently, an LC-MS/MS method using NEM derivatization has been developed and validated for the analysis of GSH and GSSG in porcine hepatocytes [16] and the assessment of the thiol redox metabolome in blood, urine, and saliva, employing a Waters Xevo TQ-S triple quadrupole mass spectrometer [17]. Tomin et al. [18] also reported an LC-MS/MS-based protocol, employing an AB Sciex 4000 QTRAP^®^ mass spectrometer, for the measurement of GSH and GSSG in blood, tissue, and cultured cells in a single analysis. In that protocol, GSH was derivatized with NEM, the reagent was removed and GSSG was reduced to GSH by TCEP (Tris (2-carboxyethyl) phosphine) and then derivatized by d5-NEM (*N*-ethyl-d5-maleimide) to generate GSH-d5-NEM. Thus, GSSG and GSH were detected as GSH-d5-NEM and GSH-NEM by LC-MS/MS with the addition of ^15^C_2_,^15^N-GSH-d5-NEM as an internal standard. The sensitivity of GSSG detection was found to be about 10-fold better than direct GSSG measurement and the samples were diluted before they were subjected to LC-MS/MS analysis. However, the method requires elaborate sample preparation, including two steps of NEM derivatization, protein precipitation, TCEP reduction, as well as the removal of the excess NEM after the first derivatization step. For the analysis of a large number of samples, a simpler and faster procedure is beneficial. 

Here, we report an optimized and simple method for the in-situ derivatization of GSH in cell culture that involves the incubation of cells in PBS-buffered NEM before methanolic extraction of GS-NEM, GSSG, and other metabolites of interest. In addition, we explored the coupling of liquid chromatographic separation to on-line UV absorbance and selected ion monitoring on a QTOF-MS instrument that may lack in contrast to a triple quadrupole mass spectrometer [16,17,18] the required linear range for the simultaneous quantification of GSH as GS-NEM and GSSG, but allows for a far more comprehensive analysis of the metabolome without having to set transitions for known metabolites of interest. Finally, we tested the applicability of the method by demonstrating the impact of genetic ablation of the monocarboxylate transporters *MCT1* and *MCT4* on the GSH/GSSG ratio of colon cancer cells under oxidative stress as well as the effect that NADPH consuming neomorphic mutations in the isocitrate dehydrogenases *IDH1/2* have on the reduction of GSSG to GSH.

## 2. Results and Discussion

### 2.1. Chromatography and Mass Spectrometry

**Figure 1:**
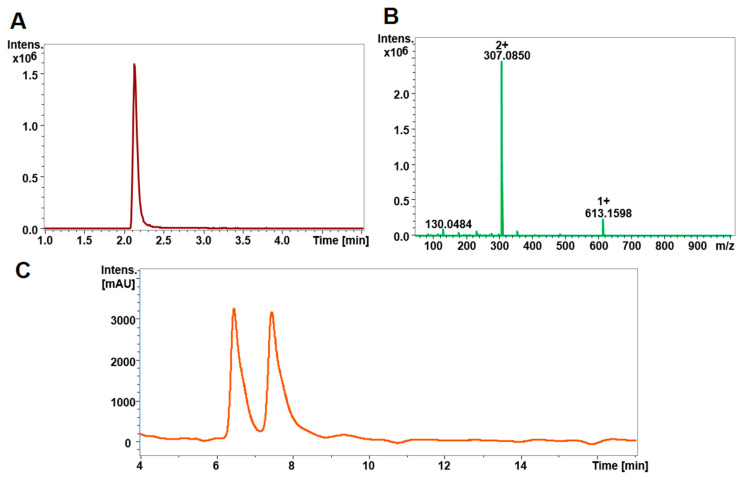
Chromatographic separation and detection of GSSG and GS-NEM. (**A**) Extracted ion chromatogram (XIC) and (**B**) mass spectrum of GSSG standard measured by HPLC-ESI-QTO-FMS. (**C**) GS-NEM was measured by HPLC-UV and the trace at 210 nm is shown. The doubly charged ion of GSSG at *m/z* 307 in Figure 1B was used for GSSG determination throughout the study. In Figure 1C, two separate GS-NEM peaks at 6.7 min and 7.8 min, respectively, were observed due to the generation of diastereomers. The peak at 6.7 min was chosen for GS-NEM determination.

Figure 1A shows an extracted ion chromatogram of a GSSG standard measured by HPLC-ESI-TOF-MS and the respective spectrum is given in Figure 1B. Figure 1C displays the UV chromatogram of a GS-NEM standard. GSSG shows a quasi-molecular ion at *m/z* 613.160 and a doubly charged ion at *m/z* 307.085. The latter yielded the higher intensity and was used for quantification. To improve the sensitivity of GSSG determination by mass spectrometry, a 10 Da Q1 selection window was employed so that only a limited *m/z* range covering unlabeled and stable isotope-labeled GSSG was transmitted and detected. Compared to full scan detection, this led to a highly significant increase in the signal-to-noise ratio of the GSSG peak (Supplementary Figure S1). GS-NEM yielded two separate peaks of equal peak area ratio with an RSD of 3.13% at 6.7 min and 7.8 min due to the generation of diastereomers that are separated under the given chromatographic conditions (Supplementary Figure S2). Here, the peak eluting at 6.7 min was used for GS-NEM quantification. GS-NEM was also detected by QTOF-MS. No other biomolecules existing in the samples coeluted with GS-NEM as evidenced by Supplementary Figure S3 demonstrating that a pooled cell sample and a GS-NEM standard share the same MS spectrum at 6.7 min. Intracellular GSH and GSSG amounts may differ by more than three orders of magnitude. Thus, simultaneous MS analysis of both glutathione forms will require an instrument with a linear range that covers four orders of magnitude, such as a triple quadrupole instrument. However, with the QTOF instrument used in this study, the response for GS-NEM was only linear up to a concentration of 62.5 µM. However, typical extract concentrations of GS-NEM were around 200 µM. Even modification of the MS parameters used for GS-NEM detection did not extend the linear range sufficiently (Supplementary Figure S4). Hence, GSSG and GSH would have to be determined separately after appropriate dilution of the sample. Thus, here, MS detection was only used to monitor the potential influences arising from the complex cell samples and UV absorbance was employed to quantify GS-NEM. 

### 2.2. Method Validation

**Figure 2:**
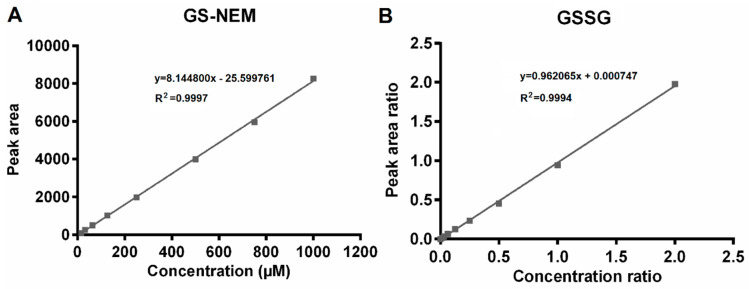
Calibration curves for GS-NEM and GSSG. (**A**) A nine-point GS-NEM calibration curve was generated over a concentration range of 15.63 µM to 1000 µM by plotting the peak area versus the corresponding nominal concentration. (**B**) A twelve-point GSSG calibration curve was constructed over a concentration range of 0.0098 µM to 10 µM based on the peak area ratios and concentration ratios of unlabeled to stable isotope-labeled GSSG (GSSG-(glycine-^13^C_4_,^15^N_2_)).

For GSSG determination, the limits of detection and quantification were 0.001 µM and 0.0098 µM, respectively. Compared to previously published methods as shown in Table 1, the here presented method features better detection sensitivity for GSSG. A twelve-point calibration covering a concentration range of 0.0098 µM to 10 µM yielded excellent linearity (*R^2^* = 0.9994). For GS-NEM determination, a nine-point calibration curve was generated, the linear range of which extended from 15.63 µM to 1000 µM (*R^2^* = 0.9997). The LOD for GS-NEM was 7.81 µM. Representative calibration curves for both GSSG and GS-NEM are shown in Figure 2. 

It should be noted that high amounts of GSSG were observed in GSH standard stock solutions after storage for one month at –20 °C (data not shown). To generate a reliable GS-NEM calibration curve, GSH stock solutions should be either freshly prepared from powder or the concentration must be recalculated through the quantification of GSSG in the stock solution.

Within-run precision was evaluated by ten successive injections of a pooled cell culture sample. The obtained peak areas of GS-NEM and the peak area ratios of GSSG to GSSG internal standard are shown in Supplementary Figure S5. The corresponding coefficients of variation (CV) for within-run repeatability of GS-NEM and GSSG were 3.48% and 3.11%, respectively. Inter-run repeatability was determined by injecting aliquots of the same pooled cell culture sample on five successive days in triplicate each day (see Supplementary Figure S6). A CV of 2.51% and 3.66% was obtained for GS-NEM and GSSG, respectively. 

**Figure 3:**
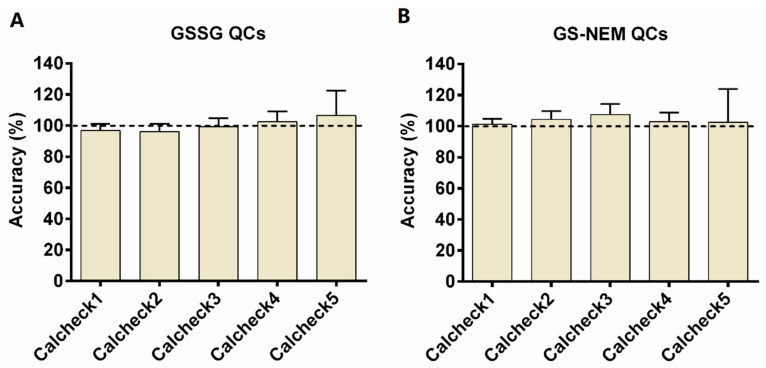
Accuracies of five quality control samples measured on different days. QCs were standard samples prepared from different stock solutions (*n* = 3) on different days (*n* = 5) within five months. (**A**) The concentrations of calcheck1 to calcheck5 for GSSG were 3 µM, 1.5 µM, 0.15 µM, 0.05 µM, and 0.02 µM, respectively. (**B**) The corresponding concentrations for calcheck1 to calcheck5 for GS-NEM were 500 µM, 200 µM, 100 µM, 50 µM, and 20 µM.

Quality control samples (QCs) of GSSG and GS-NEM were prepared from stock solutions prior to the analysis on five different days over a period of five months (see Figure 3). All QC samples showed an accuracy in the range of 80–120%. For GSSG, the respective accuracies were 96.74 ± 4.54% (calcheck1), 96.16 ± 5.16% (calcheck2), 99.27 ± 5.34% (calcheck3), 102.46 ± 6.60% (calcheck4), and 106.47 ± 16.00% (calcheck5). The corresponding accuracies for GS-NEM were 101.20 ± 3.40% (calcheck1), 104.29 ± 5.40% (calcheck2), 107.46 ± 6.68% (calcheck3), 105.32 ± 6.58% (calcheck4), and 103.79 ± 19.83% (calcheck5). Calcheck5, the closest to the LLOQ, featured the highest standard deviations of 16.00% and 19.83%, respectively, for GSSG and GS-NEM. However, mean accuracies of 106.47% and 103.79% for GSSG and GS-NEM, respectively, were still acceptable.

**Figure 4:**
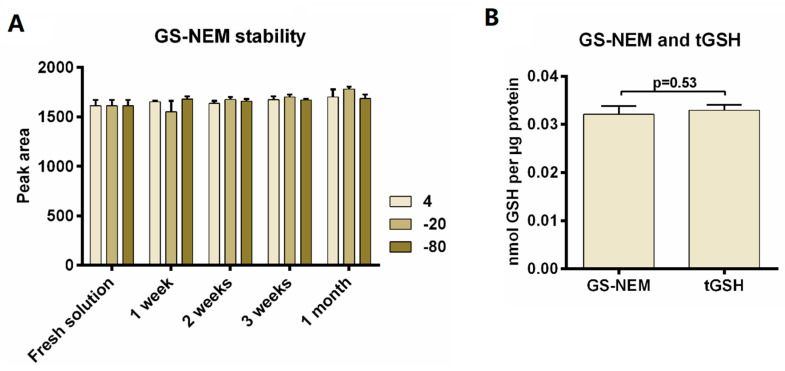
GS-NEM standard solution stability test and comparison of GS-NEM determination with total glutathione (tGSH) quantification in LS174T wild type cells. (**A**) GS-NEM stability was investigated by comparing GS-NEM peak areas after storage at different temperature for up to one month (*n* = 3). (**B**) Total GSH was measured after DTT reduction in a separate set of samples and both tGSH and GS-NEM were normalized to protein amount. No significant difference was observed between GS-NEM and tGSH (*p* = 0.53, *n* = 3).

Furthermore, we investigated the stability of GS-NEM solutions under different storage conditions. A standard GS-NEM sample was stored at 4 °C, −20 °C, and −80 °C, respectively, for the periods of time indicated in Figure 4A. The CVs of average peak area over all injections (15 injections in total) were 3.10% (4 °C), 5.66% (−20 °C), and 2.47% (−80 °C), respectively, thus attesting to adequate sample stability over at least one month.

### 2.3. Cell Harvesting

The timing of the addition of NEM to cultured cells is critical for the accurate determination of GSH as is evident from Supplementary Figure S7. The amount of GSSG determined decreased dramatically by adding NEM already during cell harvesting instead of adding it later to the methanolic cell extract. This impressively shows the importance of immediately trapping GSH to prevent autooxidation when analyzing GSSG in cultured cells.

To further optimize the protocol, we tested four different cell harvesting procedures:

Procedure 1: As described above, the cell culture medium was removed, and the cells were washed twice for 1 min with PBS containing 1 mM NEM prior to cell harvesting in cold 80% methanol.
Procedure 2: Cells were washed twice with 1 mL of PBS prior to addition of 400 µL of 1 mM NEM solution for 5 min, followed by addition of 1600 µL methanol (to a final concentration of 80%, *v/v*) to harvest cells in cold 80% methanol.
Procedure 3: Cells were washed twice with 1 mL of PBS and then scrapped with 1 mL of cold 80% methanol containing 0.5 mM NEM.
Procedure 4: NEM was added directly to the cell culture medium at a final concentration of ~1.5 mM for 2 min. Then, culture medium was discarded, cells were washed with PBS and scraped in 80% cold methanol.

Cells were seeded at the same density and each procedure was performed in triplicate. Results are shown in Supplementary Figure S8. No significant difference between the four procedures was observed for GS-NEM (ANOVA *p* = 0.59). However, a significant lower GSSG amount was detected when cells were washed with PBS containing 1 mM NEM (procedure 1), indicating that autooxidation was kept to a minimum. In procedure 4, NEM was added directly to the cell culture medium prior to PBS washing. Components present in the medium may compete for or interfere with the derivatization of GSH with NEM, which may be overcome by higher concentrations of NEM, and influence the reaction efficiency. Consequently, procedure 1 became the standard protocol. Besides, no free GSH was detected in cell culture samples after NEM derivatization (data not shown), indicating sufficient derivatization of GSH with NEM. Of note, the reaction rate of GSH with NEM in methanol is fairly low compared to the reaction in aqueous solution. Using standard samples, we observed that more than 40 min of incubation were necessary when the reaction was performed in 80% methanol. In contrast, the reaction is complete within minutes in aqueous solution. 

Using the colorectal adenocarcinoma cell line LS174T, we also compared the quantification of GSH as GS-NEM with the determination of total reduced GSH (tGSH) in a separate set of samples to validate the GS-NEM method. Quantification of tGSH employing DTT reduction was performed according to our previously reported method [21]. Briefly, cell extracts were generated using 80% methanol extraction, followed by DTT reduction to obtain total glutathione in reduced form and the analysis of tGSH by HPLC-QTOF-MS/MS. The results are shown in Figure 4B. There is no significant difference between GS-NEM and tGSH amount after normalization to protein amount. This indicates correct analysis of GSH by HPLC-UV after NEM derivatization, as the very low intracellular amounts of GSSG will not contribute significantly to tGSH. 

**Figure 5:**
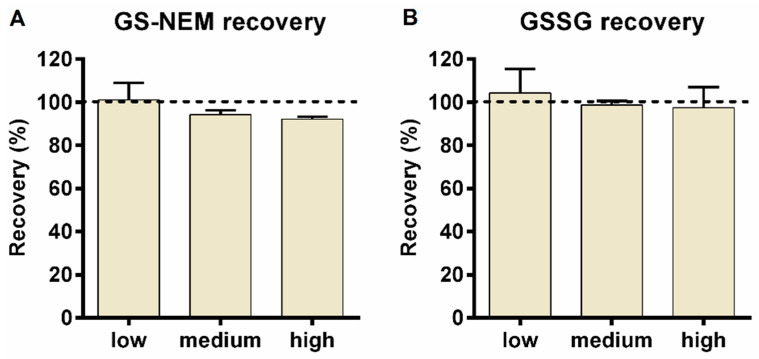
(**A**) GS-NEM and (**B**) GSSG spike-in experiments. Recovery was determined by adding defined amounts of GS-NEM or GSSG at low (10 nmol of GS-NEM, 0.02 nmol of GSSG), medium (20 nmol of GS-NEM, 0.06 nmol of GSSG), and high (40 nmol of GS-NEM, 0.2 nmol of GSSG) concentration into LS174T wild type cell cultures before 80% methanol cell extraction. GS-NEM and GSSG recovery experiments were performed separately (*n* = 3 for each).

Furthermore, spike-in experiments were performed with LS174T wild type cells to assess GSH (GS-NEM) and GSSG recovery. To minimize autooxidation artefacts, GS-NEM rather than GSH was used for the spike-in experiments. Three different GS-NEM or GSSG standard solutions of known concentration (low, medium, and high) were added to the culture dishes and cell extraction was performed as described above. The spike-in amounts were selected according to the endogenous levels of GSH (GS-NEM) and GSSG measured previously in LS174T wild type cells, which were about 20 nmol absolute for GSH and 0.02 nmol absolute for GSSG. Based on the endogenous levels, spike-in amounts of 10, 20, and 40 nmol for GS-NEM, and 0.02, 0.06, and 0.2 nmol for GSSG were selected. For each experiment, three replicates were generated. As shown in Figure 5, recovery of GS-NEM and GSSG was satisfactory for all three spike levels. Mean recovery of GS-NEM ranged between 92.2% and 101% (101.01 ± 7.96%, 94.25 ± 2.00%, and 92.15 ± 1.06%) while the mean recovery and standard deviation for GSSG was 104.28 ± 11.18%, 98.70 ± 1.99%, and 97.49 ± 9.60%, respectively.

### 2.4. GSH and GSSG Determination in Monocarboxylate Transporter Deficient Cells

**Figure 6:**
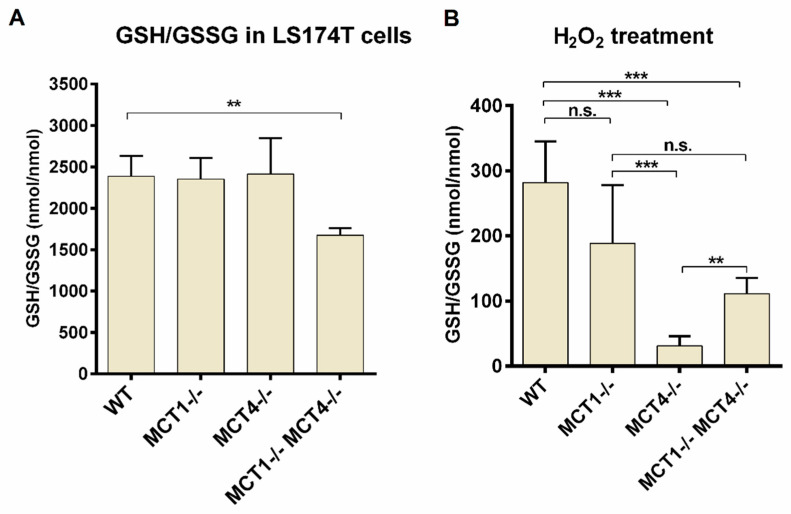
GSH/GSSG ratio in *MCT*-competent and *MCT*-deficient LS174T cells, respectively, in response to oxidative stress. Cells were (**A**) cultured under normal condition or (**B**) treated with 0.2 mM H_2_O_2_ for 10 min, before they were washed with PBS containing 1 mM NEM. Treatment with H_2_O_2_ decreases the ratio of GSH to GSSG in all cell lines. However, *MCT4^−/−^* and double knockout cells are more sensitive to oxidative stress than *MCT*-competent and *MCT1*-deficient cells. ** *p* < 0.01, *** *p*< 0.001, n.s., not significant, for further statistics see Supplementary Table S1. Detailed data presented in this figure were summarized in Supplementary Table S3.

To demonstrate the applicability of the developed HPLC-UV-QTOF-MS method, we measured the intracellular concentrations of GSH and GSSG in parental LS174T cells as well as derived single (SKO) and double knockout (DKO) clones of the monocarboxylate transporters *MCT1* and *MCT4*. As is evident from Figure 6A, under normal cell culture conditions both parental and SKO cells exhibited similar GSH/GSSG ratios with a more than 2000-fold molar excess of GSH, while the GSH/GSSG ratio of the DKO cells was significantly lower but still in excess of 1500:1. This can be readily explained by the observation [22], that complete disruption of MCT activity in LS174T cells by a combination of genetic and pharmacological means results in a more than six-fold increase in oxidative phosphorylation, which leads in turn to the increased generation of mitochondrial reactive oxygen species (ROS) and, consequently, an increase in cellular content of GSSG. As expected, when cells were challenged with 0.2 mM H_2_O_2_ for 10 min, all four cell lines showed a dramatic decrease in the GSH/GSSG ratio compared to the corresponding unstressed condition due to a collapsing NADP+/NADPH ratio (*p* < 0.001 for all cell lines, normal condition versus H_2_O_2_ treatment) (Figure 6B). However, rather unexpectedly, the decrease in GSH/GSSG ratio upon H_2_O_2_ treatment was by far the most pronounced in the *MCT4^−/−^* SKO cells (for statistics see Supplementary Table S1). 

Any attempt to interpret the above finding needs to account for the fact that both, differences in the expression of *MCT1* and *MCT4* as well as the choice of methodology to knock them out or down or to inhibit them pharmacologically may exert different effects on cell metabolism. In cells that express little, if any, *MCT4*, knockdown or pharmacological inhibition of *MCT1* has been reported to result, amongst others, in increased levels of glucose- and fructose-6-phosphate, as was observed here, as well as in marked reductions in the intracellular levels of pyruvate and GSH and in reduced glucose uptake and lactate efflux, all of which were not detected in the present study [23]. In contrast, knockdown or pharmacological inhibition of *MCT1* in cells expressing considerable amounts of *MCT4* resulted in reduced pyruvate export and increased oxygen consumption, accompanied by increased expression of genes involved in oxidative phosphorylation, while the expression of glycolytic genes such as hexokinase 1, phosphofructokinase M, and enolase 1 was decreased. Moreover, continued glucose uptake and lactate export were sustained by *MCT4* [24]. Increased mitochondrial respiration and the consequently enhanced generation of ROS are known to inactivate the M2 isozyme of pyruvate kinase (PKM2) through oxidation of Cys^358^ [25]. The resulting accumulation of phosphoenolpyruvate, in turn, results in direct catalytic inhibition of triosephosphate isomerase. This mediates a protective diversion of glucose flux into the oxidative branch of the pentose phosphate pathway (PPP) to generate NADPH required for the reduction of the antioxidants glutathione, thioredoxin and peroxiredoxin [26]. *MCT1* facilitates the proton-linked bi-directional transport of both lactate and pyruvate, while *MCT4* is considered primarily a high-affinity exporter of lactate with a significantly lower affinity for pyruvate [27]. Indeed, under unstressed conditions, growth rate adjusted export of pyruvate was lower in *MCT1^−/−^* than *MCT4^−/−^* SKO cells, while they did not differ in glucose uptake and lactate release from the parental clone (Supplementary Figure S9A–C). Given that LS174T cells express only *MCT1* and *MCT4* [22], genetic ablation of both *MCT1* and *MCT4* resulted in an almost complete inhibition of pyruvate and lactate export and very little glucose uptake, as DKO cells meet their energy requirements mostly by oxidative phosphorylation [22]. The present observation, that both the *MCT1^−/−^* SKO and the *MCT1^−/−^/MCT4^−/−^* DKO clone exhibit a higher abundance of glucose and glucose 6-phosphate (Supplementary Figure S9D) than the *MCT4^−/−^* SKO clone under unstressed conditions, may provide an important clue toward understanding the pronounced drop of GSH/GSSG ratio in the *MCT4^−/−^* SKO clone upon H_2_O_2_ treatment (Supplementary Figure S9B). As shown previously, *MCT1* blockade leads to increased mitochondrial respiration and generation of ROS, which redirect via inhibition of triosephosphate isomerase glucose flux to the PPP [22,24]. *MCT4^−/−^* null cells, in contrast, show under unstressed conditions neither a significant increase in extracellular acidification rate (ECAR) nor a significant decrease in intracellular pH [22]. The roughly two-fold increase in oxygen consumption rate (OCR) is also very modest. As cells experience an oxidative burst upon exposure to H_2_O_2_, they inactivate glycolysis within seconds via oxidation of not only pyruvate kinase but also glyceraldehyde 3-phosphate dehydrogenase, while glucose flux through the PPP continues to generate NADPH [28]. Given that glucose flux through the PPP is already increased in MCT1 deficient cells, these cells can stage most likely a faster response to H_2_O_2_ exposure, which should be reflected in lower intracellular GSSG levels compared to *MCT4^−/−^* SKO cells and *MCT1^−/−^/MCT4^−/−^* DKO cells. Indeed, as evident from Supplementary Figure S10B and D, intracellular levels of GSSG in *MCT1^−/−^* SKO cells are similar to those found in wild type cells under both unstressed and stressed conditions, with only the level of GSH being somewhat lower in *MCT1^−/−^* SKO cells under oxidative stress (Supplementary Figure S10A,C). Both *MCT4^−/−^* SKO and *MCT1^−/−^/MCT4^−/−^* DKO cells show highly significant increases in GSSG content compared to parental and *MCT1^−/−^* SKO cells under stressed condition (Supplementary Figure S10D). Interestingly, the increase in GSSG content in DKO cells as compared to *MCT* competent cells is lower than in *MCT4^−/−^* SKO cells (Supplementary Figure S10D) and further compensated by a higher GSH content in the DKO cells (Supplementary Figure S10C). In conclusion, it appears that *MCT4^−/−^* null cells are poorly adapted to sudden bursts of oxidative stress.

### 2.5. GSH and GSSG Determination in Isocitrate Dehydrogenase Wild Type and Mutant Cells

**Figure 7:**
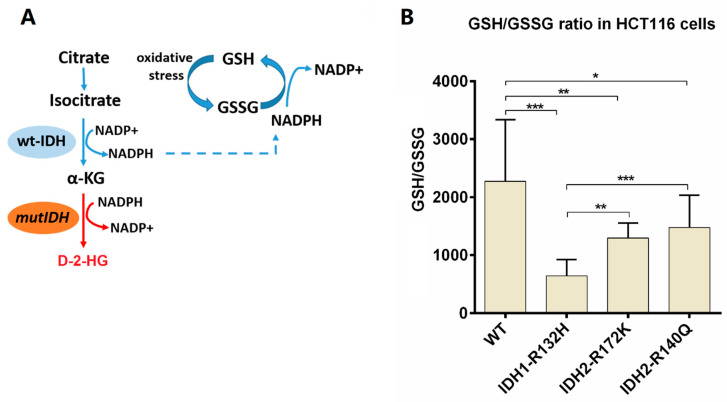
(**A**) Scheme depicting the reactions catalyzed by wild type and mutant IDH enzymes and their respective effects on the provision of NADPH for the reduction of GSSG to GSH by glutathione reductase. (**B**) Effect of different *IDH1/2* mutations on the GSH/GSSG ratio in HCT116 cells compared to *IDH1/2* wild type cells. * *p* < 0.05, ** *p* < 0.01, *** *p* < 0.001, *n* = 3, for further statistics see Supplementary Table S5.

Next, we applied the developed method to the determination of the intracellular levels of GSH and GSSG in the colon cancer cell line HCT116, in which we had already determined previously total GSH content [21]. The wild type and isocitrate dehydrogenase 1/2 (*IDH1/2*) mutant cell clones IDH1-R132H, IDH2-R172K, and IDH2-R140Q, respectively, were used to study the effect on the GSH/GSSG ratio in cells carrying neomorphic *IDH1/2* mutations, which enable cells to catalyze the NADPH consuming reduction of α-ketoglutarate (α-KG) to D-2-hydroxyglutarate (D-2-HG) (see Figure 7A) [29,30,31]. As shown in Figure 7B, all *mutIDH* cell lines show a significant lower GSH/GSSG ratio compared to the wild type cell line (for statistics, see Supplementary Table S3), supporting the notion that increased consumption of NADPH by *IDH1/2* mutant cells will impair their ability to reduce GSSG to GSH. Interestingly, the GSH/GSSG ratios observed in the three mutant cell lines appear to correlate indirectly with the amounts of D-2-hydroxyglutarate detected in these cells [32]. Furthermore, *mutIDH1* cells seem to be less capable of regenerating GSH than *mutIDH2* (mitochondrial isoform) cells. *IDH1* is the cytosolic isoform, and therefore increased consumption of NADPH by the mutated enzyme has a more direct effect on the reduction of GSSG, which also takes place in the cytosol.

## 3. Experimental

### 3.1. Chemicals and Reagents

Stable isotope-labeled glutathione disulfide (glutathione-(glycine-^13^C_4_,^15^N_2_)) was acquired from Toronto Research Chemicals (Toronto, Canada). GSH, GSSG, and NEM were purchased from Sigma Aldrich (Taufkirchen, Germany). Solvents for sample preparation and HPLC-MS analysis were HPLC grade and obtained from Th. Geyer GmbH (Renningen, Germany).

### 3.2. Stock Solutions

Stock solutions of unlabeled GSH (2 mM) and GSSG (1 mM), and stable isotope-labeled GSSG (10 mM) were prepared in purified water (PURELAB Plus system, ELGA LabWater, Celle, Germany) and stored at –20 °C. Working solutions were prepared freshly before analysis. Aliquots of NEM (310 mM) were prepared in purified water and stored at –20 °C. In-house quality controls (QCs) of five different concentration levels of GS-NEM (500 µM, 200 µM, 100 µM, 50 µM, and 20 µM) and GSSG (3 µM, 1.5 µM, 0.15 µM, 0.05 µM, and 0.02 µM) were also prepared freshly.

### 3.3. Cell Culture and Sample Preparation

LS174T wild type and *MCT1/4* single and double knockout clones [22] as well as HCT116 wild type and *IDH* mutant cells (Horizon Discovery Ltd., Cambridge, UK) were cultivated in RPMI (PAN, Aidenbach, Germany), supplemented with 10% FCS (Biochrom AG, Berlin, Germany), 1% penicillin-streptomycin (PAA Laboratories Inc., Pasching, Austria), and 2 mM L-glutamine (PAA). Cells were seeded in 6-well plates in triplicates at a density of 450,000 cells per well and incubated overnight at 37 °C, 5% CO_2_. 

To harvest cells for GSSG and GSH analysis, the medium was discarded, and the cells were washed twice for 1 min each with 1 mL PBS (PAN) containing 1 mM NEM. Then, 10 µL of 25 µM GSSG internal standard were added to each well before cells were scrapped in 600 µL of cold 80% methanol. The extract was transferred to a 1.5 mL cup and the wells were washed with 400 µL cold 80% methanol. The wash was collected into the same cup. Samples were then stored overnight at −80 °C. 

The collected cell extracts were centrifuged at 4 °C and 10,000× *g* for 5 min. The supernatants were collected, and the pellets were washed twice with 200 µL 80% methanol. The combined supernatants were evaporated to dryness (CombiDancer, Hettich AG, Bach, Switzerland) and then re-dissolved in 50 µL pure water before HPLC-UV-QTOF-MS analysis.

### 3.4. HPLC-UV-QTOFMS Analysis of GSH and GSSG

Instrumental analysis was carried out on a Maxis Impact QTOF-MS (Bruker Daltonics, Bremen, Germany) with an ESI source coupled to a Dionex Ultimate 3000 UHPLC system (Thermo Scientific, Idstein, Germany) consisting of a HPG3400 RS pump, WPS3000TFC autosampler, and a Dionex diode-array detector. Chromatographic separation was performed on a Waters Atlantis T3 reversed-phase column (2.1 × 150 mm, 3 µm) fitted with a 2.0 × 4 mm C18 pre-column (Phenomenex, Aschaffenburg, Germany). Mobile phases A and B were 0.1% (*v/v*) formic acid in H_2_O and acetonitrile, respectively. Gradient elution was started with a 15 min isocratic segment at 95% A, before ramping B from 5% to 100% in 2 min, followed by a 3 min hold and re-equilibration at 95% A for 5 min. The column temperature was set at 35 °C and a flow rate of 0.3 mL/min was used. Samples were injected in random order with an injection volume of 10 µL.

For GS-NEM determination, the diode-array detector was operated over a range of 200 to 400 nm. The GS-NEM absorption peak was extracted at 210 nm. The eluent from the diode-array detector was subsequently transferred to the QTOF mass spectrometer via an ESI source for GSSG detection in positive mode using either full scan 50 to 1000 *m/z* or a 10 Da Q1 selection window. GSSG quantification was achieved using a 12-point calibration curve based on the area ratios of unlabeled to stable isotope-labeled compound. GS-NEM quantification was performed using a calibration curve generated from the HPLC-UV measurements of a serial dilution of a GS-NEM standard solution. MS parameters and mass calibration were as recently reported [21]. For internal recalibration, each run was started with injection of a sodium formate solution [21] by a six-port valve. Mass spectrometry detection was divided into different segments for the separate monitoring of sodium formate clusters, GSSG, and GS-NEM.

Determination of lactate, pyruvate, glucose, and glucose-6-phosphate was performed as described in Supplementary File S1.

### 3.5. Method Validation

#### 3.5.1. Figures of Merit

The linear range for GSSG quantification was determined based on a serial dilution of a GSSG standard (10 µM to 0.0024 µM) with a constant concentration of the internal standard (5 µM). The calibration curve was built based on the peak area ratio of analyte to internal standard versus the corresponding nominal concentration ratio. The lower limit of quantification (LLOQ) and limit of detection (LOD) were defined according to the FDA Guide for Bioanalytical Method Validation [33] with LLOQ as the lowest calibration curve concentration, for which the analyte can be quantitatively determined with an accuracy of 80–120%, and the LOD as the lowest analyte concentration that yields a peak with S/N ≥ 3.

Linearity of GS-NEM quantification was evaluated in a concentration range of 15.63 µM to 1000 µM. A GS-NEM standard solution was produced by the reaction of fresh GSH standard with NEM in the lab. Calibration samples were diluted from a standard GS-NEM solution. 

#### 3.5.2. Stability of GS-NEM

Stability of GS-NEM derivative was assessed by comparing peak areas obtained for a 200 µM GS-NEM standard solution stored at 4 °C, −20 °C, and −80 °C for different time periods.

#### 3.5.3. Recovery

Recovery of GS-NEM and GSSG was assessed separately by spiking low, medium, and high amounts of GS-NEM (10 nmol, 20 nmol, concentration in the stock solution was recalculated by the quantification of GSSG with the 40 nmol) or GSSG (0.02 nmol, 0.06 nmol, 0.2 nmol) into cell cultures prior to sample preparation. Recovery of GS-NEM and GSSG was determined separately to avoid the interference of GSSG present in GSH standard solution due to auto-oxidation. GSH addition of stable isotope labeled GSSG internal standard. Furthermore, blank samples, cell samples without addition of GS-NEM or GSSG, were analyzed to determine endogenous GSH and GSSG amounts, which were subtracted from the values determined in the spiked samples for recovery calculation. Using GSSG as an example, recovery was determined as follows:

$$\mathrm{Recovery}=\frac{GSSG\;amount\;in\;spiked\;sample-GSSG\;amount\;in\;blank\;sample}{theoretical\;amount\;of\;GSSG\;standard\;spiked}\times 100\%$$

Absolute amounts were normalized to protein amount in each sample to correct for differences in cell number. All experiments were performed in triplicate.

### 3.6. Data Analysis and Statistics

Data analysis was carried out using Bruker QuantAnalysis 2.2 software. Statistical differences between more than 2 groups were tested using single factor ANOVA with Tukey’s post-hoc HSD test in R version 3.5.1, while differences between two groups were tested by the unpaired two-tailed *t*-test implemented in Excel 2013. A value of *p* < 0.05 was considered statistically significant. Results are presented as mean plus standard deviation (M+SD). Figures were prepared with GraphPad Prism 6.

## 4. Conclusions

The analysis of GSH/GSSG ratio in cell culture is a challenging task due to auto-oxidation of GSH during cell harvesting and metabolite extraction. Here, we have optimized both the in-situ derivatization of GSH with NEM and the simultaneous determination of GSH and GSSG by HPLC-UV and LC-QTOF-MS in cultured cells. The method is rapid and shows high sensitivity, excellent precision, and nearly complete recovery of both GSH and GSSG in spike-in experiments.

## Figures and Tables

**Table 1:** Comparison of methods reported for the determination of GSH and GSSG.

Method	Sample	GSH LOD *	GSH LOQ *	GSSG LOD *	GSSG LOQ *	Derivatization	Ref.
Enzymatic recycling	Rat liver/bile	--	6.25 pmol	--	2.17 pmol	M4VP	[19]
HPLC-UV	Erythrocytes	820 pmol (0.041 mM)	2700 pmol (0.135 mM)	--	--	DTNB	[20]
HPLC-fluorescence	Plasma	0.6 pmol (0.03 µM)	2 pmol (0.10 µM)	--	--	NBD-F	[8]
LC-MS/MS	Whole blood	4 pmol (0.4 µM)	15 pmol (1.5 µM)	1.5 pmol (0.1 µM)	1.5 pmol (0.1 µM)	NEM	[13]
HPLC-UV	Cultured cells	--	--	--	--	NEM/DTT/mBrB	[15]
HPLC-UV	Cultured cells	78.1 pmol (7.81 µM)	156.5 pmol (15.65 µM)	--	--	NEM	This study
HPLC-QTOF-MS		--	--	0.01 pmol	0.1 pmol	--	

M4VP: 1-methyl-4-vinyl-pyridinium; DTNB: 5,5′ -dithio-bis-(2-nitrobenzoic acid); NBD-F: 7-flouro-4-nitrobenzo-2-oxa-1,3-diazole; NEM: *N*-ethylmaleimide; mBrB: monobromobimane; DTT: dithiothreitol. * Amount of substance loaded on column. LODs and LOQs presented in each study were converted to the same unit and shown as amount of substance loaded on column by multiplying the concentration of the analyte with the injection volume in each study. The concentrations shown in the brackets are the original values presented in each study.

## Supplementary Materials

The following are available online at https://www.mdpi.com/2218-1989/10/7/292/s1, Figure S1: Comparison of signal-to-noise ratio for 2.5 µM solution of GSSG measured by mass spectrometry in full scan or MRM mode (*n* = 3). Figure S2: Peak area comparison of GS-NEM diastereomers analyzed by HPLC-UV. Diastereomers eluted at 6.7 min and 7.8 min, respectively. Peak area ratios of the GS-NEM diastereomers in cultured cell samples are all stably around 1 with an RSD of 3.13%. Figure S3: Exemplary MS spectra of GS-NEM detected by QTOF-MS in (A) standard sample and (B) pooled cell sample. GS-NEM shows a [M+H]+ ion at *m/z* 433 and a fragment ion at *m/z* 304 due to the loss of Glu. Figure S4: (A) Extracted ion chromatograms of quasi-molecular ion (*m/z* 433.14) of GS-NEM (31.25 µM) detected by mass spectrometry with different parameter settings. (B) Extracted ion chromatogram of quasi-molecular ion (*m/z* 433.14) of GS-NEM (250 µM) analyzed by LC-QTOF-MS. Detection saturation of GS-NEM was still a problem with each setting. In our experiments, GS-NEM concentrations detected in cell extracts were mostly higher than 200 µM. However, as shown in Figure S4B, detection saturation is severe when a GS-NEM solution with a concentration of 250 µM was analyzed, indicating the necessary dilution of the samples for MS detection. Figure S5: (A) Peak areas of GS-NEM and (B) peak area ratios of GSSG to GSSG internal standard for ten successive injections of a pooled cell culture sample measured by HPLC-UV-QTOF-MS. Figure S6: (A) Peak areas of GS-NEM and (B) peak area ratios of GSSG to GSSG internal standard (GSSG is) for a pooled cell culture sample measured on 5 successive days by HPLC-UV-QTOFMS (*n* = 3 for each day). Figure S7: Peak area of GSSG detected in cell culture samples after derivatization of GSH with NEM either in the final extract or during cell harvesting by adding NEM to the PBS wash (*n* = 3). Figure S8: Optimization of the NEM derivatization procedure. Procedure 1, cell medium was discarded, followed by two 1-min washing steps with PBS containing 1 mM NEM. Procedure 2, cell medium was discarded, followed by PBS washing twice. Then, 400 µL of 1 mM NEM was added to the cells and incubated at room temperature for 5 min before harvesting the cells in 80% methanol. Procedure 3, cells were harvested with 1 mL of 80% methanol containing 0.5 mM NEM after PBS washing twice. Procedure 4, 10 µL of 310 mM NEM were added directly to the cells and incubated for 2 min before discarding the medium and PBS washing, *n* = 3 for each procedure. No significant difference in GS-NEM amount was observed between groups (ANOVA, *p* = 0.59). For GSSG, significant differences were found between groups (ANOVA, *p* = 0.0068): 1 versus 2: *p* = 0.0364; 1 versus 3: *p* = 0.0171; 1 versus 4: *p* = 0.0064. One-way ANOVA and post hoc analysis with Tukey’s test were performed in R (version 3.5.1). Figure S9: Release of (A) pyruvate and (B) lactate as well as uptake of glucose normalized to (C) area under the growth curve, and (D) intracellular content of glucose and glucose 6-phosphate (G6P) in unstressed LS174T parental and MCT1/4 single and double knockout clones cultured for 24 h. Metabolites in methanolic extracts of both cell culture supernatants (A, B) and cell pellets (D) were analyzed by GC-MS. Two independent experiments (*n* = 6, three for each experiment) were performed. (ANOVA for pyruvate *p* = 5.1 × 10^-4^, for lactate secretion *p* = 9.7 × 10^-9^, for glucose uptake *p* = 1.3 × 10^-9^, for intracellular glucose *p* = 2.4 × 10^-6^ and intracellular G6P *p* = 2.8×10^-5^, * *p* < 0.05, ** *p* < 0.01, *** *p* < 0.001, n.s., not significant). For further statistics see Table S2. GC-MS analysis of lactate, pyruvate, glucose and glucose 6-phosphate, protein content determination, and cell proliferation rate determination see above experimental section. Figure S10: Intracellular levels of GSH (A and C) and GSSG (B and D) in LS174T parental and MCT1/4 single and double knockout clones before (unstressed, *n* = 6 each) and after treatment with 0.2 mM H_2_O_2_ for 10 min (*n* = 3 each). One-way ANOVA (*p* = 0.0473 for GSH, unstressed; *p* = 7.44 × 10^-7^ for GSSG, unstressed; *p* = 1.65 × 10^-4^ for GSH, H_2_O_2_ treated; *p* = 2.04 × 10^-6^ for GSH, H_2_O_2_ treated) and post hoc analysis with Tukey’s test were performed in R (version 3.5.1). * *p* < 0.05, ** *p* < 0.01, *** *p* < 0.001. Detailed data presented in this figure were summarized in Table S4. Table S1: Analysis of variance (ANOVA) of GSH/GSSG ratio between MCT-competent and MCT-deficient LS174T cells under normal or H_2_O_2_ treatment conditions was performed in R (version 3.5.1). Pairwise comparisons between cell lines under each condition were performed with Tukey’s post hoc test. A paired *t*-test (EXCEL 2013) was used to test the impact of H_2_O_2_ treatment in each cell line. A *p*-value of less than 0.05 was statistically significant. n.s., not significant. Table S2: Analysis of variance (ANOVA) of pyruvate secretion, lactate release, glucose uptake, and intracellular glucose and G6P content between MCT-competent and MCT-deficient LS174T cells under normal conditions were performed in R (version 3.5.1). Pairwise comparisons between cell lines under each condition were performed with Tukey’s post hoc test. A *p*-value of less than 0.05 was statistically significant. n.s., not significant. Table S3: Extract concentrations of GSH and GSSG from LS174T cells with/without H_2_O_2_ treatment. Data are shown in Figure 6. Table S4: GSH and GSSG amounts in LS174T cells with/without H_2_O_2_ treatment after normalization to total protein. Data are presented in Figure S9. Table S5: Analysis of variance (ANOVA) of GSH/GSSG ratios between HCT116 cell lines was performed in R (version 3.5.1) with Tukey’s post hoc test. A *p*-value of less than 0.05 was considered statistically significant. n.s., not significant. Supplementary File S1.
