# Supplementary material for: Optimized Protocol for the In Situ Derivatization of Glutathione with N-Ethylmaleimide in Cultured Cells and the Simultaneous Determination of Glutathione/Glutathione Disulfide Ratio by HPLC-UV-QTOF-MS
Source: Metabolites. 2020 Jul 17;10(7):292. doi: 10.3390/metabo10070292 (PMC7407321; doi:10.3390/metabo10070292)
Supplement: Supplementary file 1 [file metabolites-10-00292-s001.zip › Supplementary/Figure and Table.docx]

Optimized protocol for the *In Situ* derivatization of glutathione with N-ethylmaleimide in cultured cells and the simultaneous determination of the glutathione/ glutathione disulfide ratio by HPLC-UV-QTOF-MS

Xueni Sun^1^, Raffaela S Berger^1^, Paul Heinrich^1^, Ibtissam Marchiq^2^, Jacques Pouyssegur^2,3^, Kathrin Renner^4^, Peter J Oefner^1^, Katja Dettmer^1*^

^*^Corresponding author

^1^Institute of Functional Genomics, University of Regensburg, Am BioPark 9, 93053 Regensburg, Germany

^2^University Côte d’Azur (IRCAN), CNRS-INSERM, Centre A. Lacassagne, 06189, Nice, France

^3^Department of Medical Biology, Centre Scientifique de Monaco, CSM, 98000 Monaco, Monaco

^4^Department of Internal Medicine III, University Hospital Regensburg, 93042 Regensburg, Germany





**Figure S1**. Comparison of signal-to-noise ratio for 2.5 µM solution of GSSG measured by mass spectrometry in full scan or MRM mode (n=3).


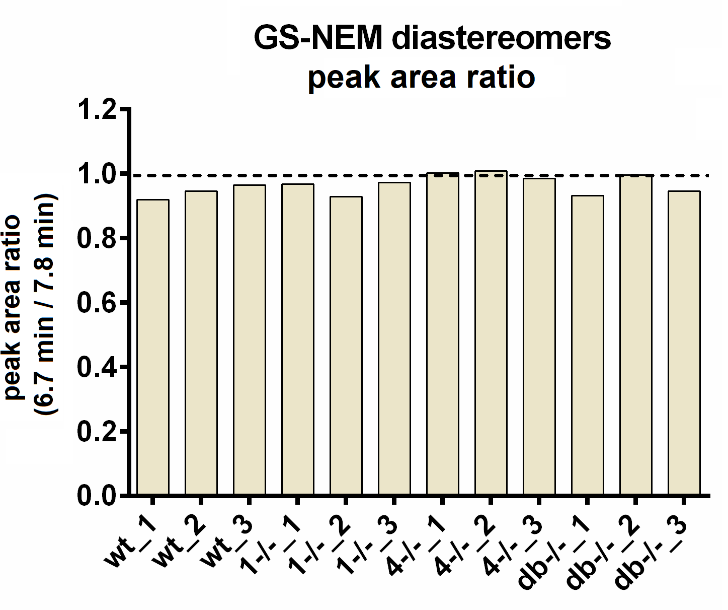


**Figure S2**. Peak area comparison of GS-NEM diastereomers analyzed by HPLC-UV. Diastereomers eluted at 6.7 min and 7.8 min, respectively. Peak area ratios of the GS-NEM diastereomers in cultured cell samples are all stably around 1 with an RSD of 3.13%.


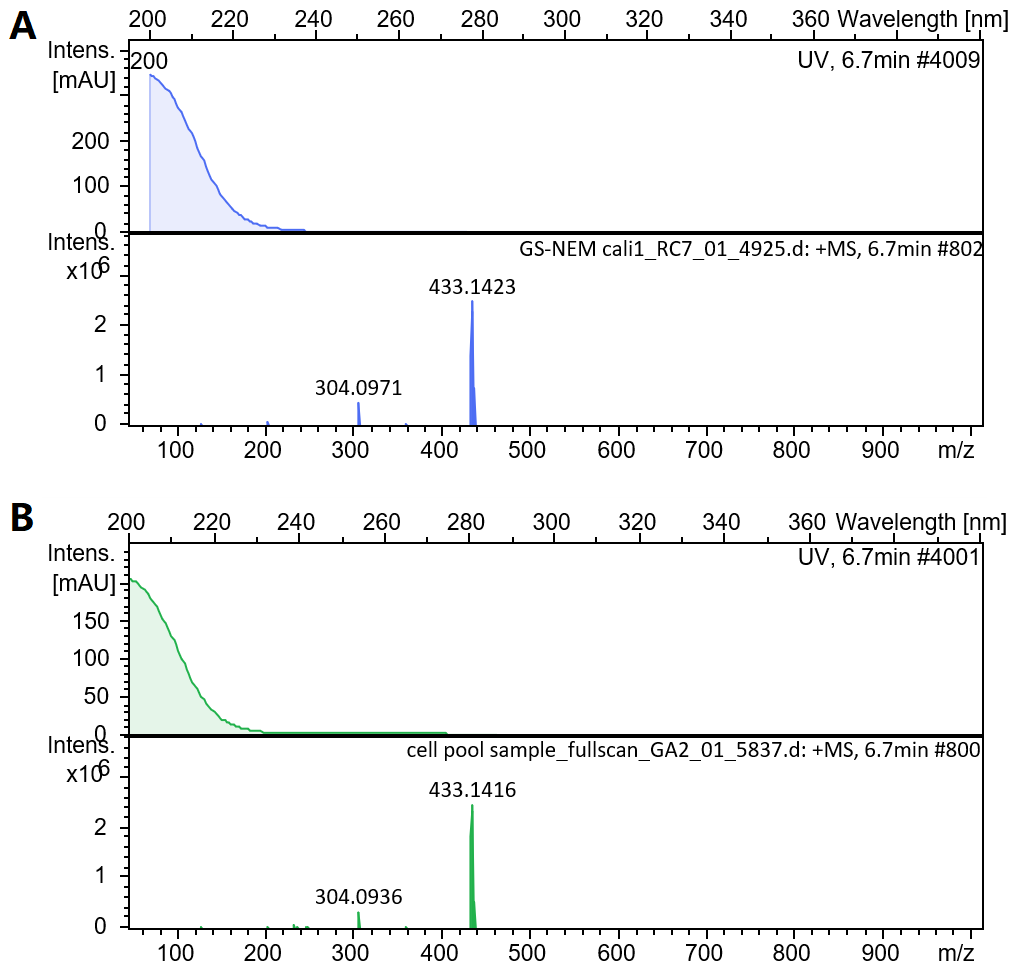


**Figure S3**. Exemplary MS spectra of GS-NEM detected by QTOF-MS in (A) standard sample and (B) pooled cell sample. GS-NEM shows a [M+H]^+^ ion at *m/z* 433 and a fragment ion at *m/z* 304 due to the loss of Glu.


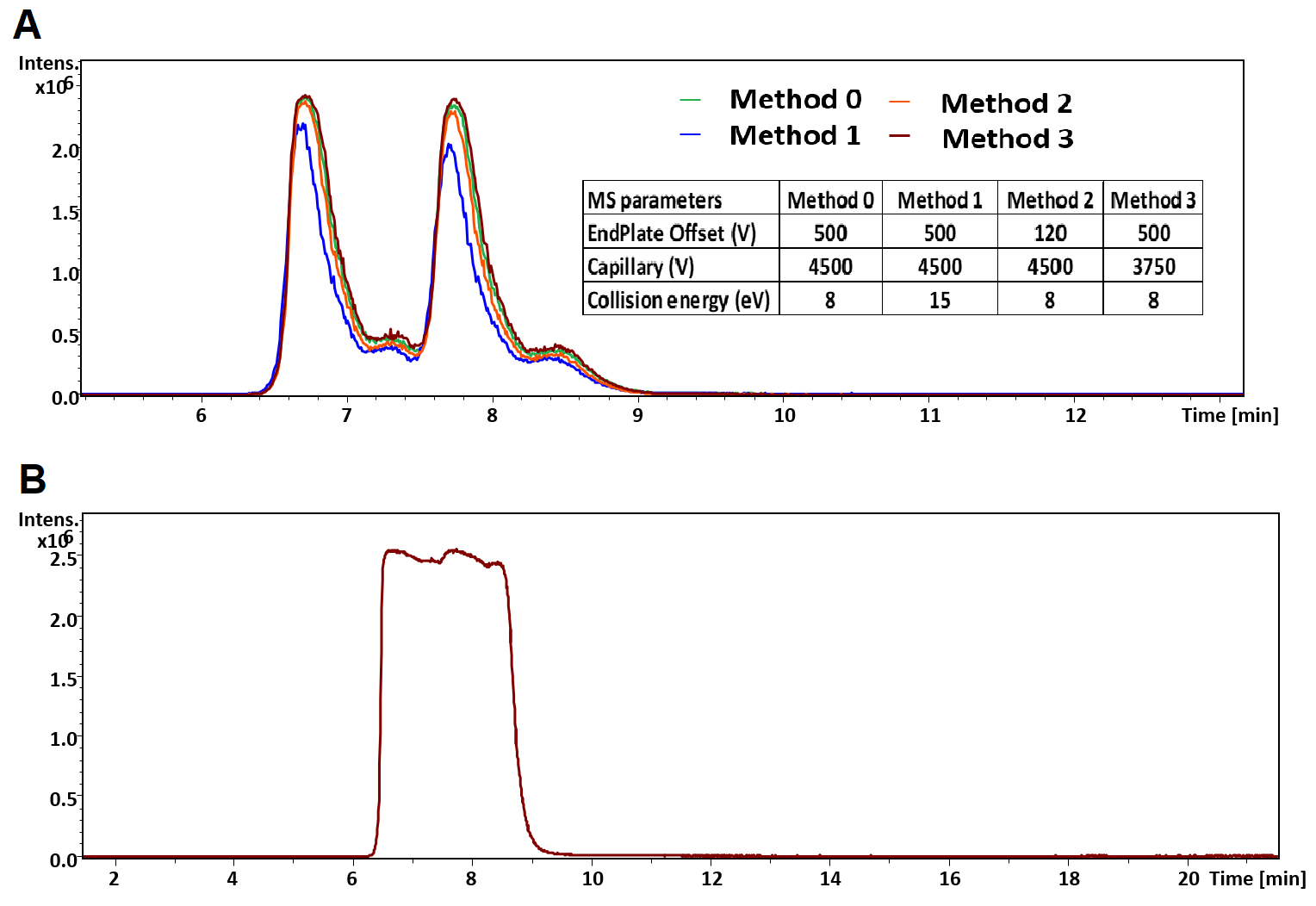


**Figure S4**. (A) Extracted ion chromatograms of quasi-molecular ion (*m/z* 433.14) of GS-NEM (31.25 µM) detected by mass spectrometry with different parameter settings. (B) Extracted ion chromatogram of quasi-molecular ion (*m/z* 433.14) of GS-NEM (250 µM) analyzed by LC-QTOFMS. Detection saturation of GS-NEM was still a problem with each setting. In our experiments, GS-NEM concentrations detected in cell extracts were mostly higher than 200 µM. However, as shown in Figure S4B, detection saturation is severe when a GS-NEM solution with a concentration of 250 µM was analyzed, indicating the necessary dilution of the samples for MS detection.


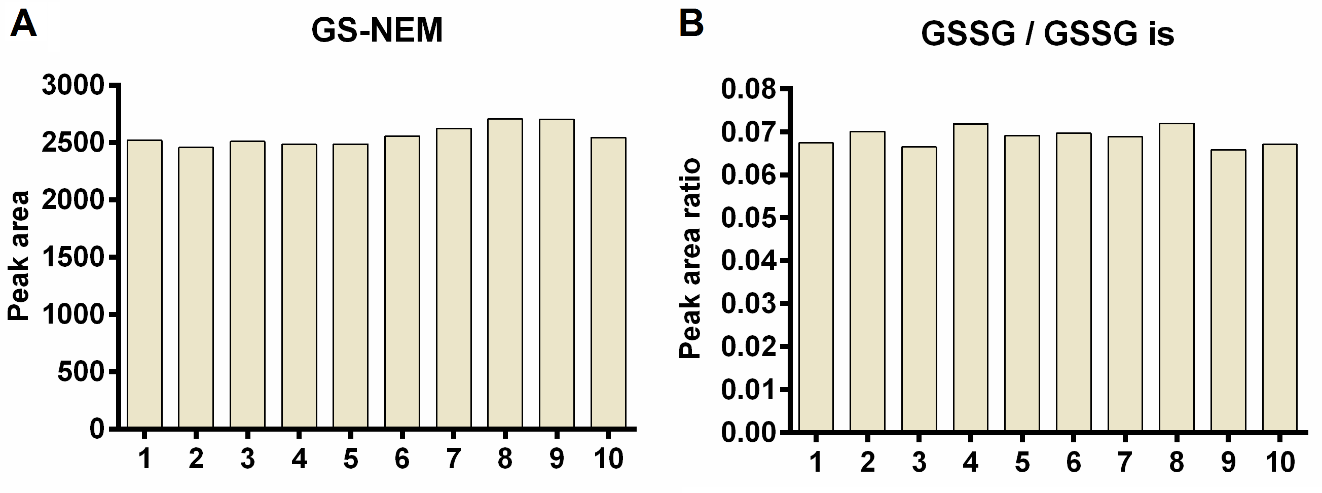


**Figure S5**. (A) Peak areas of GS-NEM and (B) peak area ratios of GSSG to GSSG internal standard for ten successive injections of a pooled cell culture sample measured by HPLC-UV-QTOF-MS.


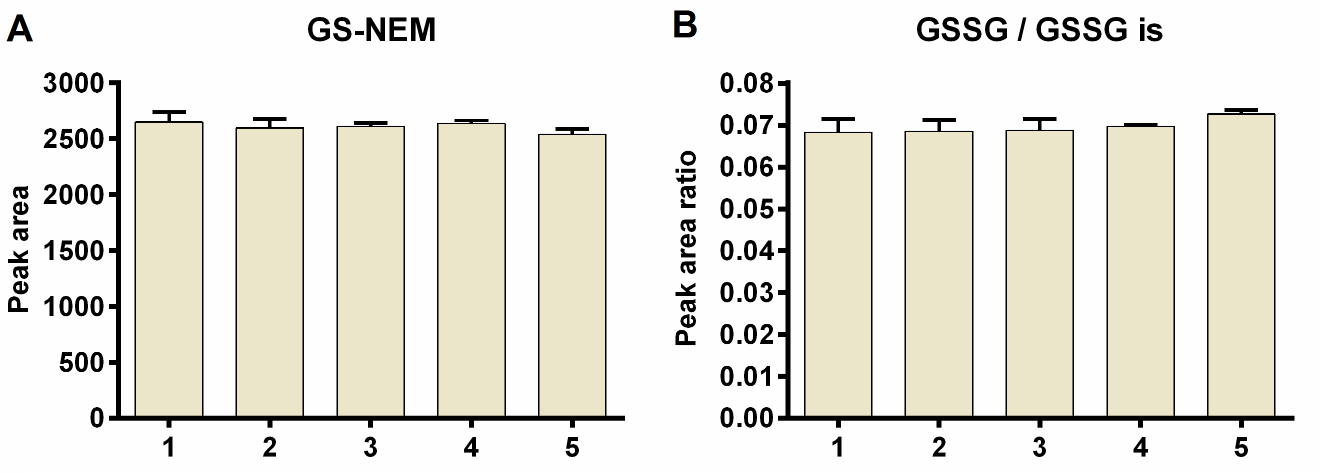


**Figure S6**. (A) Peak areas of GS-NEM and (B) peak area ratios of GSSG to GSSG internal standard (GSSG is) for a pooled cell culture sample measured on 5 successive days by HPLC-UV-QTOF-MS (n=3 for each day).


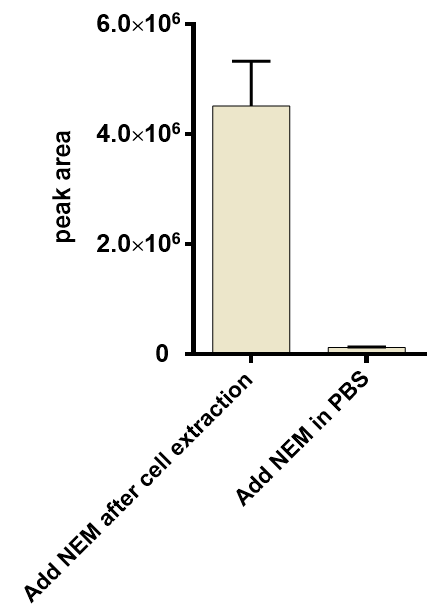


**Figure S7**. Peak area of GSSG detected in cell culture samples after derivatization of GSH with NEM either in the final extract or during cell harvesting by adding NEM to the PBS wash (n=3).


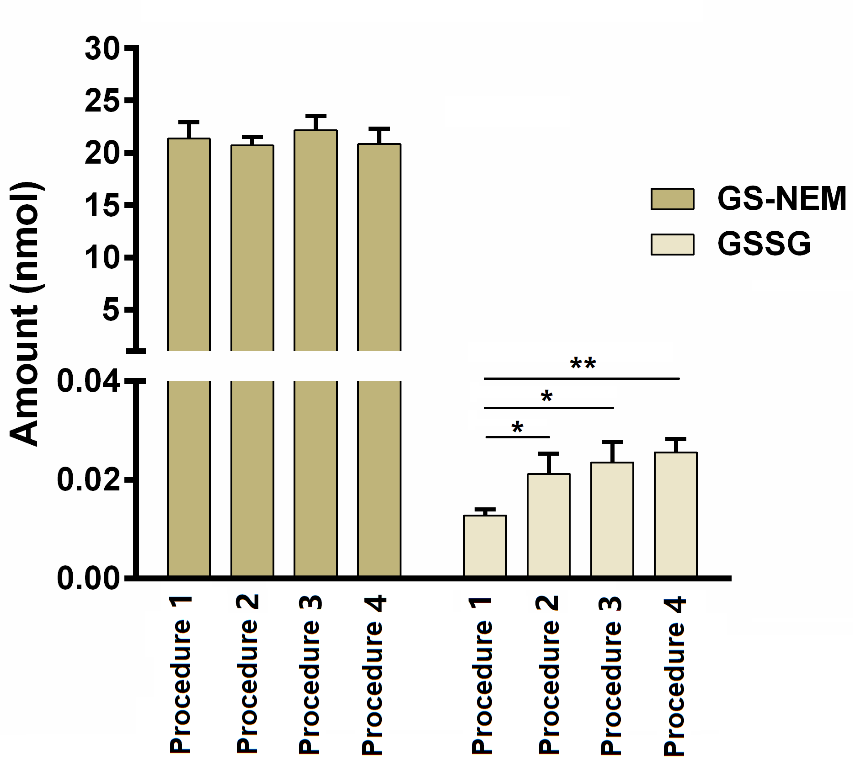


**Figure S8**. Optimization of the NEM derivatization procedure. **Procedure 1**, cell medium was discarded, followed by two 1-min washing steps with PBS containing 1 mM NEM. **Procedure 2**, cell medium was discarded, followed by PBS washing twice. Then, 400 µL of 1 mM NEM was added to the cells and incubated at room temperature for 5 min before harvesting the cells in 80% methanol. **Procedure 3**, cells were harvested with 1 mL of 80% methanol containing 0.5 mM NEM after PBS washing twice. **Procedure 4**, 10 µL of 310 mM NEM were added directly to the cells and incubated for 2 min before discarding the medium and PBS washing, n=3 for each procedure. No significant difference in GS-NEM amount was observed between groups (ANOVA, p=0.59). For GSSG, significant differences were found between groups (ANOVA, p=0.0068): 1 versus 2: p=0.0364; 1 versus 3: p=0.0171; 1 versus 4: p=0.0064. One-way ANOVA and post hoc analysis with Tukey’s test were performed in R (version 3.5.1).


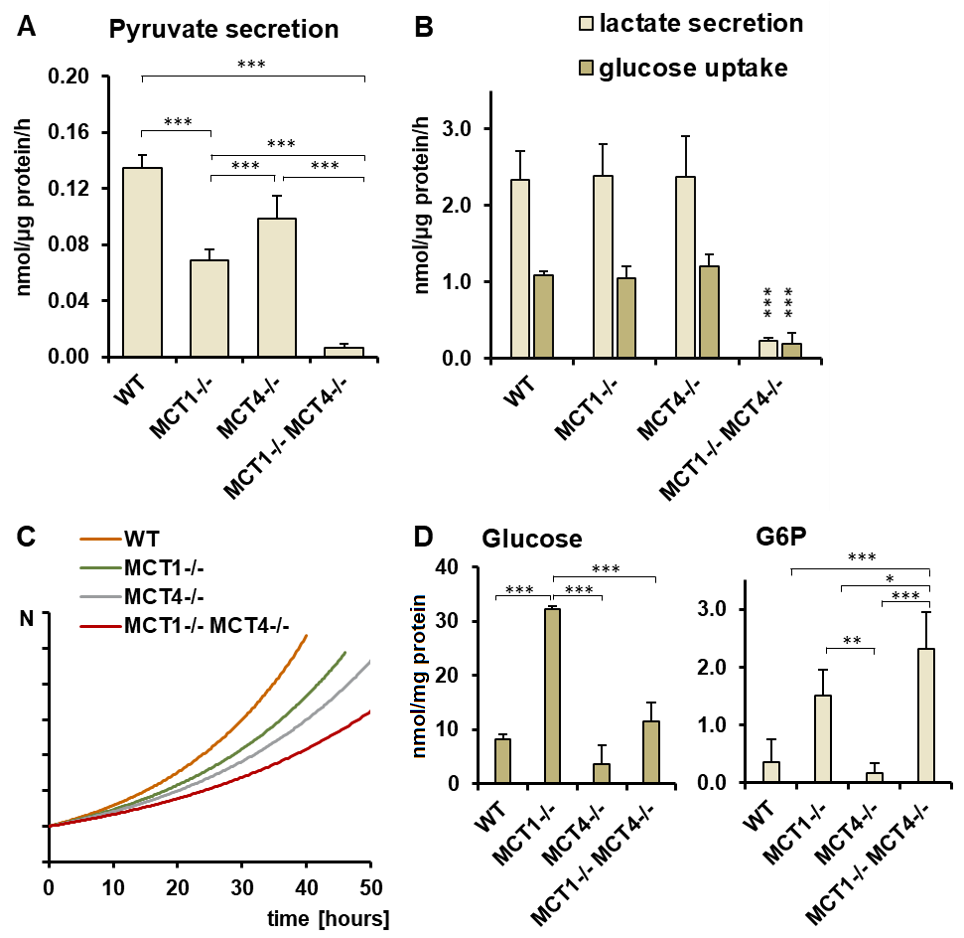


**Figure S9:** Release of (A) pyruvate and (B) lactate as well as uptake of glucose normalized to (C) area under the growth curve, and (D) intracellular content of glucose and glucose 6-phosphate (G6P) in unstressed LS174T parental and MCT1/4 single and double knockout clones cultured for 24 h. Metabolites in methanolic extracts of both cell culture supernatants (A, B) and cell pellets (D) were analyzed by GC-MS. Two independent experiments (n=6, three for each experiment) were performed. (ANOVA for pyruvate p = 5.1×10^-4^, for lactate secretion p = 9.7×10^-9^, for glucose uptake p = 1.3×10^-9^, for intracellular glucose p = 2.4×10^-6^ and intracellular G6P p = 2.8×10^-5^, * p< 0.05, ** p< 0.01, *** p< 0.001, n.s., not significant). For further statistics see Table S2. GC-MS analysis of lactate, pyruvate, glucose and glucose 6-phosphate, protein content determination, and cell proliferation rate determination see above experimental section.


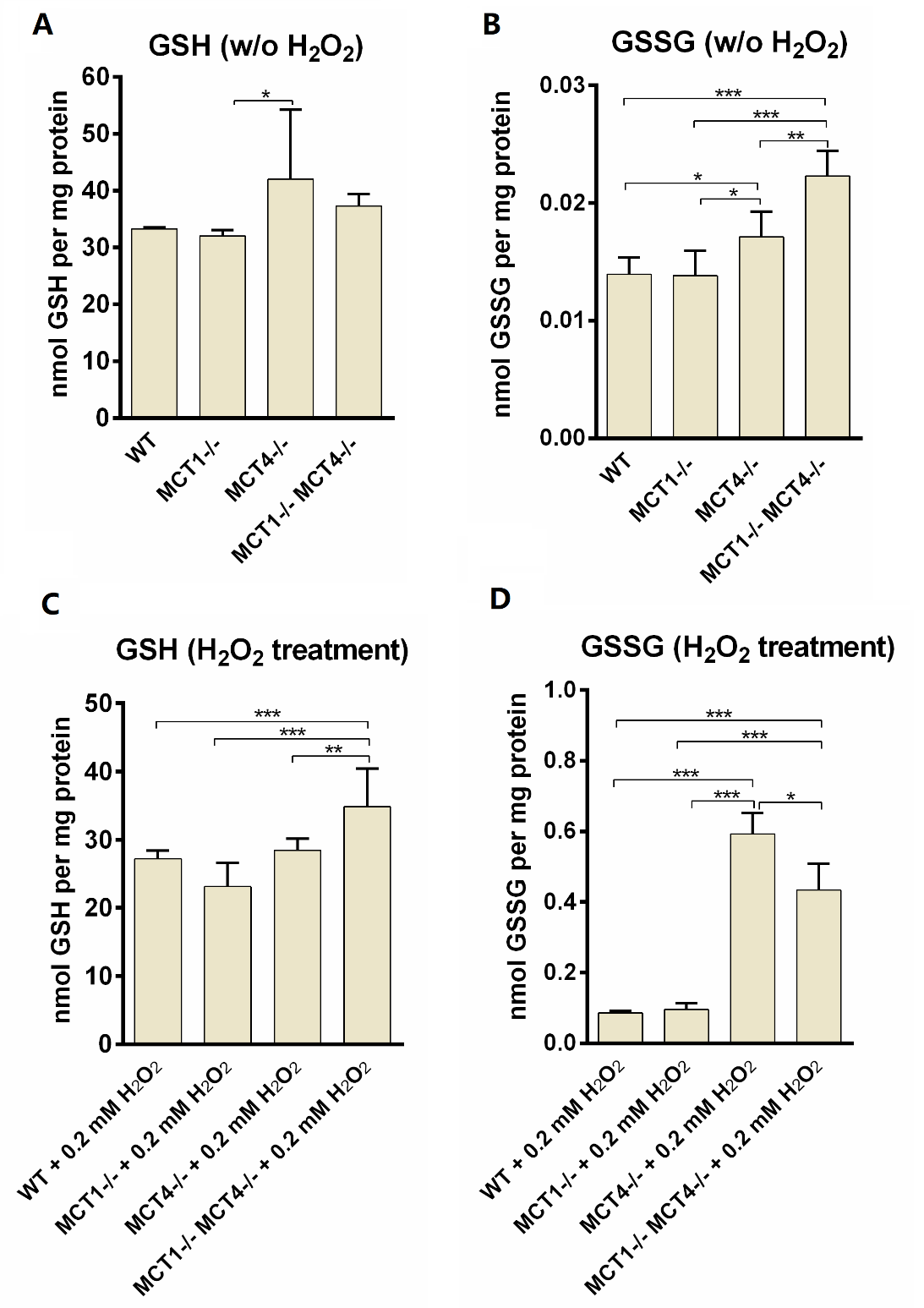


**Figure S10.** Intracellular levels of GSH (A and C) and GSSG (B and D) in LS174T parental and *MCT1/4* single and double knockout clones before (unstressed, n=6 each) and after treatment with 0.2 mM H_2_O_2_ for 10 min (n=3 each). One-way ANOVA (p=0.0473 for GSH, unstressed; p=7.44×10^-7^ for GSSG, unstressed; p=1.65×10^-4^ for GSH, H_2_O_2_ treated; p=2.04×10^-6^ for GSH, H_2_O_2_ treated) and post hoc analysis with Tukey’s test were performed in R (version 3.5.1). * p< 0.05, ** p< 0.01, *** p< 0.001. Detailed data presented in this figure were summarized in Table S4.

**Table S1**. Analysis of variance (ANOVA) of GSH/GSSG ratio between MCT-competent and MCT-deficient LS174T cells under normal or H_2_O_2_ treatment conditions was performed in R (version 3.5.1). Pairwise comparisons between cell lines under each condition were performed with Tukey’s post hoc test. A paired t-test (EXCEL 2013) was used to test the impact of H_2_O_2_ treatment in each cell line. A p-value of less than 0.05 was statistically significant. n.s., not significant.

| **Normal condition (overall p-value = 0.0004)** | | | | |
| --- | --- | --- | --- | --- |
|  | WT | *MCT1-/-* | *MCT4-/-* | *MCT1-/- MCT4-/-* |
| WT | -- | -- | -- | -- |
| *MCT1-/-* | n.s. | -- | -- | -- |
| *MCT4-/-* | n.s. | n.s. | -- | -- |
| *MCT1-/- MCT4-/-* | 0.0016 | 0.0027 | 0.0011 | -- |
| **H_2_O_2_ treatment (overall p-value =** **2.13×10^-6^)** | | | | |
|  | WT | *MCT1-/-* | *MCT4-/-* | *MCT1-/- MCT4-/-* |
| WT | -- | -- | -- | -- |
| *MCT1-/-* | n.s. | -- | -- | -- |
| *MCT4-/-* | 1.50×10^-6^ | 7.04×10^-4^ | -- | -- |
| *MCT1-/- MCT4-/-* | 2.22×10^-4^ | n.s. | 0.0014 | -- |
| **Normal condition versus H_2_O_2_ treatment** | | | | |
|  | WT | *MCT1-/-* | *MCT4-/-* | *MCT1-/- MCT4-/-* |
| WT | 8.69×10^-6^ | -- | -- | -- |
| *MCT1-/-* | -- | 7.90×10^-6^ | -- | -- |
| *MCT4-/-* | -- | -- | 3.61×10^-5^ | -- |
| *MCT1-/- MCT4-/-* | -- | -- | -- | 1.05×10^-7^ |

**Table S2.** Analysis of variance (ANOVA) of pyruvate secretion, lactate release, glucose uptake, and intracellular glucose and G6P content between MCT-competent and MCT-deficient LS174T cells under normal conditions were performed in R (version 3.5.1). Pairwise comparisons between cell lines under each condition were performed with Tukey’s post hoc test. A p-value of less than 0.05 was statistically significant. n.s., not significant.

|  | **Under normal condition (overall p-value = 2.86x10^-13^) – pyruvate secretion** | | | | | | |
| --- | --- | --- | --- | --- | --- | --- | --- |
|  |  | WT | *MCT1-/-* | | *MCT4-/-* | *MCT1-/- MCT4-/-* | |
|  | WT | -- | -- | | -- | -- | |
|  | *MCT1-/-* | 1.33x10^-8^ | -- | | -- | -- | |
|  | *MCT4-/-* | 9.38x10^-5^ | 6.86x10^-4^ | | -- | -- | |
|  | *MCT1-/- MCT4-/-* | 2.28x10^-13^ | 1.33x10^-8^ | | 5.77x10^-11^ | -- | |
|  | **Under normal condition (overall p-value = 9.67x10^-9^) – lactate release** | | | | | | |
|  |  | WT | *MCT1-/-* | | *MCT4-/-* | *MCT1-/- MCT4-/-* | |
|  | WT | -- | -- | | -- | -- | |
|  | *MCT1-/-* | n.s | -- | | -- | -- | |
|  | *MCT4-/-* | n.s | n.s | | -- | -- | |
|  | *MCT1-/- MCT4-/-* | 1.57x10^-7^ | 5.33x10^-8^ | | 1.19x10^-7^ | -- | |
|  | **Under normal condition (overall p-value = 1.29x10^-9^) – glucose uptake** | | | | | | |
|  |  | WT | *MCT1-/-* | | *MCT4-/-* | *MCT1-/- MCT4-/-* | |
|  | WT | -- | -- | | -- | -- | |
|  | *MCT1-/-* | n.s | -- | | -- | -- | |
|  | *MCT4-/-* | n.s. | n.s. | | -- | -- | |
|  | *MCT1-/- MCT4-/-* | 2.70x10^-8^ | 4.67x10^-8^ | | 3.81x10^-9^ | -- | |
|  | **Under normal condition (overall p-value = 2.43x10^-6^) – intracellular glucose** | | | | | | |
|  |  | WT | *MCT1-/-* | | *MCT4-/-* | | *MCT1-/- MCT4-/-* |
|  | WT | -- | -- | | -- | | -- |
|  | *MCT1-/-* | 2.01x10^-5^ | -- | | -- | | -- |
|  | *MCT4-/-* | n.s. | 3.80x10^-6^ | | -- | | -- |
|  | *MCT1-/- MCT4-/-* | n.s. | 2.90x10^-4^ | | n.s. | | -- |
|  | **Under normal condition (overall p-value = 2.81x10^-5^) – intracellular G6P** | | | | | | |
|  |  | WT | | *MCT1-/-* | *MCT4-/-* | *MCT1-/- MCT4-/-* | |
|  | WT | -- | | -- | -- | -- | |
|  | *MCT1-/-* | n.s. | | -- | -- | -- | |
|  | *MCT4-/-* | n.s. | | 4.72x10^-3^ | -- | -- | |
|  | *MCT1-/- MCT4-/-* | 6.14x10^-4^ | | 1.62x10^-2^ | 2.54x10^-5^ | -- | |

**Table S3.** Extract concentrations of GSH and GSSG from LS174T cells with / without H_2_O_2_ treatment. Data are shown in Figure 6.

|  | **W/O H_2_O_2_** | | | **H_2_O_2_** | | |
| --- | --- | --- | --- | --- | --- | --- |
| **Experiment #1** | **GSH (µM)** | **GSSG (µM)** | **GSH/GSSG** | **GSH (µM)** | **GSSG (µM)** | **GSH/GSSG** |
| 1-/-_1 | 248.0067 | 0.0940 | 2637.79 | 210.9651 | 0.6721 | 313.90 |
| 1-/-_2 | 250.9572 | 0.1053 | 2383.38 | 209.7773 | 0.9341 | 224.58 |
| 1-/-_3 | 260.7391 | 0.1177 | 2216.09 | 208.0241 | 1.0188 | 204.19 |
| 4-/-_1 | 518.0444 | 0.1867 | 2775.00 | 469.3140 | 10.7161 | 43.80 |
| 4-/-_2 | 518.4150 | 0.1824 | 2841.72 | 495.0983 | 9.2155 | 53.72 |
| 4-/-_3 | 514.6896 | 0.1840 | 2796.55 | 424.3565 | 9.0215 | 47.04 |
| db-/-_1 | 146.9669 | 0.0837 | 1755.80 | 628.6451 | 5.5561 | 113.14 |
| db-/-_2 | 161.6730 | 0.0952 | 1698.69 | 641.9056 | 6.7050 | 95.74 |
| db-/-_3 | 155.7395 | 0.0943 | 1651.82 | 570.1374 | 7.9238 | 71.95 |
| WT_1 | 293.3980 | 0.1117 | 2627.29 | 247.4414 | 0.7002 | 353.40 |
| WT_2 | 279.3835 | 0.1058 | 2641.80 | 235.9096 | 0.8254 | 285.80 |
| WT_3 | 296.4558 | 0.1239 | 2393.32 | 231.4389 | 0.7201 | 321.42 |
| **Experiment #2** | **GSH (µM)** | **GSSG (µM)** | **GSH/GSSG** | **GSH (µM)** | **GSSG (µM)** | **GSH/GSSG** |
| 1-/-_1 | 283.2138 | 0.1308 | 2165.28 | 274.1929 | 1.2703 | 215.84 |
| 1-/-_2 | 274.2360 | 0.1342 | 2043.37 | 264.6429 | 4.1374 | 63.96 |
| 1-/-_3 | 249.5782 | 0.0937 | 2663.62 | 247.1810 | 2.2513 | 109.80 |
| 4-/-_1 | 411.9132 | 0.2012 | 2047.61 | 543.7372 | 20.3093 | 26.77 |
| 4-/-_2 | 469.4007 | 0.2223 | 2111.70 | 474.4560 | 37.9692 | 12.50 |
| 4-/-_3 | 355.5441 | 0.1862 | 1909.85 | 433.3557 | 17.4250 | 24.87 |
| db-/-_1 | 513.8810 | 0.2923 | 1757.79 | 527.2954 | 3.8562 | 136.74 |
| db-/-_2 | 513.6130 | 0.3098 | 1658.15 | 565.5199 | 4.7041 | 120.22 |
| db-/-_3 | 529.6641 | 0.3448 | 1536.36 | 631.8866 | 4.8599 | 130.02 |
| WT_1 | 266.0126 | 0.1076 | 2471.45 | 266.2900 | 1.5989 | 166.54 |
| WT_2 | 264.7406 | 0.1263 | 2096.21 | 254.1800 | 0.9138 | 278.14 |
| WT_3 | 281.7083 | 0.1346 | 2092.23 | 244.3313 | 0.8549 | 285.81 |

**Table S4.** GSH and GSSG amounts in LS174T cells with / without H_2_O_2_ treatment after normalization to total protein. Data are presented in Figure S9.

|  | **W/O H_2_O_2_** | | **H_2_O_2_** | |
| --- | --- | --- | --- | --- |
| **Experiment #1** | **GSH  (nmol per µg protein)** | **GSSG (nmol per µg protein)** | **GSH  (nmol per µg protein)** | **GSSG (nmol per µg protein)** |
| 1-/-_1 | 31.3195 | 0.0119 | 24.4287 | 0.0778 |
| 1-/-_2 | 31.2541 | 0.0131 | 25.7629 | 0.1147 |
| 1-/-_3 | 31.6132 | 0.0143 | 19.1850 | 0.0940 |
| 4-/-_1 | 52.8833 | 0.0191 | 28.9458 | 0.6609 |
| 4-/-_2 | 53.9501 | 0.0190 | 29.8035 | 0.5547 |
| 4-/-_3 | 51.6859 | 0.0185 | 26.4688 | 0.5627 |
| db-/-_1 | 33.3889 | 0.0190 | 41.9510 | 0.3708 |
| db-/-_2 | 36.3395 | 0.0214 | 39.6055 | 0.4137 |
| db-/-_3 | 37.7602 | 0.0229 | 37.1254 | 0.5160 |
| WT_1 | 33.3943 | 0.0127 | 28.0464 | 0.0794 |
| WT_2 | 33.5140 | 0.0127 | 25.7860 | 0.0902 |
| WT_3 | 32.9605 | 0.0138 | 27.6689 | 0.0861 |
| **Experiment #2** | **GSH  (nmol per µg protein)** | **GSSG (nmol per µg protein)** | **GSH  (nmol per µg protein)** | **GSSG (nmol per µg protein)** |
| 1-/-_1 | 34.1956 | 0.0158 | -- | -- |
| 1-/-_2 | 33.7770 | 0.0165 | -- | -- |
| 1-/-_3 | 29.9689 | 0.0113 | -- | -- |
| 4-/-_1 | 30.9367 | 0.0151 | -- | -- |
| 4-/-_2 | 35.7232 | 0.0169 | -- | -- |
| 4-/-_3 | 26.7351 | 0.0140 | -- | -- |
| db-/-_1 | 38.9680 | 0.0222 | -- | -- |
| db-/-_2 | 37.6408 | 0.0227 | -- | -- |
| db-/-_3 | 39.4073 | 0.0256 | -- | -- |
| WT_1 | 32.4434 | 0.0131 | -- | -- |
| WT_2 | 31.5187 | 0.0150 | -- | -- |
| WT_3 | 33.9438 | 0.0162 | -- | -- |

**Table S5**. Analysis of variance (ANOVA) of GSH/GSSG ratios between HCT116 cell lines was performed in R (version 3.5.1) with Tukey`s post hoc test. A p-value of less than 0.05 was considered statistically significant. n.s., not significant.

| **overall p-value = 0.0004** | | | | |
| --- | --- | --- | --- | --- |
|  | WT | IDH1-R132H | IDH2-R172K | IDH2-R140Q |
| WT | -- | -- | -- | -- |
| IDH1-R132H | 0.0004 | -- | -- | -- |
| IDH2-R172K | 0.0040 | 0.0021 | -- | -- |
| IDH2-R140Q | 0.0106 | 0.0004 | n.s. | -- |

1. Jain, M.; Nilsson, R.; Sharma, S.; Madhusudhan, N.; Kitami, T.; Souza, A.L.; Kafri, R.; Kirschner, M.W.; Clish, C.B.; Mootha, V.K. Metabolite profiling identifies a key role for glycine in rapid cancer cell proliferation. *Science* **2012**, *336*, 1040-1044.

2. Dettmer, K.; Nurnberger, N.; Kaspar, H.; Gruber, M.A.; Almstetter, M.F.; Oefner, P.J. Metabolite extraction from adherently growing mammalian cells for metabolomics studies: optimization of harvesting and extraction protocols. *Anal Bioanal Chem* **2011**, *399*, 1127-1139.
