# Supplementary material for: Optimized Protocol for the In Situ Derivatization of Glutathione with N-Ethylmaleimide in Cultured Cells and the Simultaneous Determination of Glutathione/Glutathione Disulfide Ratio by HPLC-UV-QTOF-MS
Source: Metabolites. 2020 Jul 17;10(7):292. doi: 10.3390/metabo10070292 (PMC7407321; doi:10.3390/metabo10070292)
Supplement: Supplementary file 1 [file metabolites-10-00292-s001.zip › Supplementary/Supplementary File S1.docx]

## Experimental section:

## Protein content determination:

Intracellular metabolite concentrations were normalized to total protein content determined by the FluoroProfile® Protein Quantification Kit (Sigma-Aldrich) according to manufacturer’s instructions or using the fluorescent dye SERVA Purple (SERVA, Heidelberg, Germany) in the same manner. Briefly, cell pellets were lysed in a sodium phosphate buffer (20 mM) with 1.2% SDS. Samples were diluted, if necessary, with water and subjected to fluorometric analysis at excitation and emission wavelengths of 485 nm and 600 nm, respectively.

#### GC-MS analysis of lactate, pyruvate, glucose and glucose 6-phosphate

Uptake of glucose and release of lactate and pyruvate were determined by GC-MS analysis cell culture supernatants of LS174T parental and *MCT1/4* knockout clones grown for 24 hours. 10 µL of cell culture supernatant were spiked with 10 µL internal standard solution containing ^13^C_3_-lactate, ^13^C_3_-pyruvate, ^13^C_6_-glucose, ^13^C_6_-glucose-6-phosphate (each 1 mM) and dried directly in a flat bottom insert in 1.5- mL vial for subsequent GC-MS analysis. The measured concentrations were converted to uptake/release data (molar amounts per mg cellular protein per unit time) by subtracting the fresh medium concentration of each respective metabolite, and normalizing to the area under the growth curve according to Jain et al. [[1](#_ENREF_1)].

For determination of the intracellular concentrations of glucose and glucose 6-phosphate by GC-MS, the cell culture medium was removed and cells were washed with 1 mL PBS twice before cell-scraping with 600 µL cold 80% methanol. During scraping, 10 μL of an internal standard solution (see above) was added to each sample. The sample suspension was collected in a 1.5-mL cup. The wells were further rinsed with 400 µL cold 80% methanol and the wash was added to the sample extract. Samples were then stored at -80 °C. Further sample extraction was performed as described above. The dried sample extract was subjected to GC-MS analysis employing the derivatization protocol and instrumental setup previously described [[2](#_ENREF_2)]. Splitless injection with an injection volume of 1 μL was performed. Quantification was achieved based on calibration curves using the corresponding stable isotope labeled analog as internal standard.

## Cell proliferation rate determination:

Proliferation rates were determined by harvesting cells at different time points from 0 h to 48 h and using the total cell protein content as a measure for cell number N (N0 and N(t), respectively). Then the doubling time τ was calculated using the following formula: $\tau= ln2/\frac{ln(\frac{N(t)}{N_{0}})}{t} .$
